# No indication for SARS-CoV-2 transmission to pet ferrets, in five cities in Poland, 2021 - antibody testing among ferrets living with owners infected with SARS-CoV-2 or free of infection

**DOI:** 10.1186/s13028-023-00672-3

**Published:** 2023-02-28

**Authors:** Edyta Kaczorek-Łukowska, Kerstin Wernike, Martin Beer, Alicja Blank, Joanna Małaczewska, Mirosława Blank, Anna Jałonicka, Andrzej Krzysztof Siwicki

**Affiliations:** 1 Department of Microbiology and Clinical Immunology, Faculty of Veterinary Medicine, University of Warmia and Mazury in Olsztyn, Oczapowskiego 13, 10-719 Olsztyn, Poland; 2 Institute of Diagnostic Virology, Friedrich-Loeffler-Institut, Südufer 10, 17493 Greifswald - Insel Riems, Germany; 3 Association of Friends of Ferrets, Mickiewicza 18a/4, 01-517 Warsaw, Poland; 4 PULSVET Specialist Veterinary Clinic, Alternatywy 7/U8, 02-775 Warsaw, Poland

**Keywords:** COVID-19, Ferret, Mustelid, Serology, Viral diseases

## Abstract

The severe acute respiratory syndrome coronavirus 2 (SARS-CoV-2) was first identified in China by the end of 2019 and was responsible for a pandemic in the human population that resulted in millions of deaths worldwide. Since the beginning of the pandemic, the role of animals as spill-over or reservoir hosts was discussed. In addition to cats and dogs, ferrets are becoming increasingly popular as companion animals. Under experimental conditions, ferrets are susceptible to SARS-CoV-2 and it appears that they can also be infected through contact with a SARS-CoV-2 positive owner. However, there is still little information available regarding these natural infections. Here, we serologically tested samples collected from pet ferrets (n = 45) from Poland between June and September 2021. Of the ferrets that were included in the study, 29% (13/45) had contact with owners with confirmed SARS-CoV-2 infections. Nevertheless, SARS-CoV-2-specific antibodies could not be detected in any of the animals, independent of the infection status of the owner. The obtained results suggest that ferrets cannot be readily infected with SARS-CoV-2 under natural conditions, even after prolonged contact with infected humans. However, due to the rapid mutation rate of this virus, it is important to include ferrets in future monitoring studies.

**Supplementary Information:**

The online version contains supplementary material available at 10.1186/s13028-023-00672-3.

## Findings

Coronaviruses are common in the environment and are able to infect and cause clinical disease in mammals (genera *Alpha* and *Beta coronavirus*) and birds (genera *Delta* and *Gamma coronavirus*) [1, 2]. These viruses adapt to new hosts, which is related to their mutation, recombination and specific replication mechanisms [3]. The course of infection depends on the infected species, and respiratory, neurological and enteric forms are described. To date, the most significant coronavirus in terms of public health worldwide is the severe acute respiratory syndrome coronavirus 2 (SARS-CoV-2) [4, 5]. This virus, which causes the human disease COVID-19, was first identified in Wuhan (China) in November 2019, and in early 2020, the World Health Organization (WHO) declared a pandemic. Although SARS-CoV-2 is considered to be of animal origin, most likely from bats, the main focus was initially on human infections, as the virus caused millions of fatalities worldwide [6, 7]. However, as the pandemic progressed and the first natural infections in animals were reported [7–9], questions arose about the role of animals and which species could be an intermediate or reservoir host. In the field, anthropozoonotic infections of felines, canines, cervids and hominids have been confirmed, but the most worrisome was the discovery that American mink (*Neovion vison*) are highly susceptible to the pathogen [10]. In the case of mink, it turned out that under natural conditions they can be infected by humans and transmit the virus to other mink and to humans [11, 12]. In light of this information, investigations into whether other animals related to mink also show such high susceptibility to SARS-CoV-2 have received interest. Ferrets, which, like mink, belong to the family *Mustelidae*, are becoming increasingly popular as companion animals due to their intelligence and sociability. Although they are still not as popular as dogs and cats, their number in households is increasing every year [13]. To date, field infections of ferrets from infected owners have been occasionally reported, e.g., in Spain, Slovenia and USA [8, 14, 15]. Here, we used serology to investigate previous SARS-CoV-2 infections in pet ferrets in Poland.

Serum samples were collected from pet ferrets (n = 45) living under the care of both, the Ferret Friends Association (n = 2) and private owners (n = 43), from different parts of Poland (Warsaw: 16; Gdansk: 7; Wroclaw: 6; Swidnica: 15 and Walbrzych: 1) between June and September 2021. The age, sex and residential area of the ferrets are given in Additional file 1. All animals were housed in cage-free homes and all animals had frequent direct contact with their owners. The owners were asked whether they had been previously infected with SARS-CoV-2 (SARS-CoV-2 real-time reverse transcription polymerase chain reaction (RT-PCR) positive result, the specific date of the test result is not known to the authors) and whether they had observed any clinical signs in their animals during the pandemic, i.e., since the beginning of 2020. At the day of sampling, all animals were clinically unsuspicious.

The selection of animals was random and based on the owner’s decision as to whether stored blood samples of their animals may be used for SARS-CoV-2 serology. The blood was originally collected at selected veterinary offices during routine health examinations of the ferrets. Blood (0.5 mL) was collected from the cephalic vein into a test tube with a clotting activator. After collection, the samples were immediately sent to the laboratory at 4 °C.

The ferret sera were tested for the presence of anti-SARS-CoV-2 antibodies by a multispecies enzyme-linked immunoassay (ELISA) based on the receptor binding domain (RBD) performed exactly as previously described [16]. The optical density (OD) was read at a wavelength of 450 nm on a Tecan Spectra Mini instrument (Tecan Group Ltd., Männedorf, Switzerland). An absorbance of ≥ 0.3 was defined as seropositive, while values of ≤ 0.2 were defined as negative by prior validation and the intermediate zone between 0.2 and 0.3 was defined as inconclusive. Known positive and negative ferret control sera were tested in parallel. The suitability of the indirect ELISA for investigation of ferret sera was proven during the initial validation using samples obtained from experimentally infected ferrets and negative control sera collected before the SARS-CoV-2 pandemic [16].

Thirteen of the 45 ferrets (29%) had contact with owners with confirmed SARS-CoV-2 infections and all of the ferrets tested negative for antibodies against SARS-CoV-2. From January 2020 until blood sampling in mid-2021, clinical signs/diseases were observed in only six animals and included pneumonia, splenomegaly, bronchitis, chronic renal failure and sneezing (Additional file 1). Nevertheless, SARS-CoV-2-specific antibodies could not be detected in any of the animals, independent of the infection status of the owner and of the observation of clinical signs (Additional file 1).

While there are several reports by their owners about natural SARS-CoV-2 infections of popular companion animal species like cats and dogs [17], the situation for pet ferrets is still largely unknown. Since ferrets are additionally used as laboratory animals in biomedical research and represent a model organism to study human infection with respiratory viruses such as influenzavirus type A, the choice of these animals for the study of the pathogenesis of SARS-CoV-2 seemed obvious [8]. Experimental infections have demonstrated that ferrets can become infected, develop weak to moderate clinical signs, shed the virus for up to 21 days after infection and produce antibodies against viral proteins including SARS-CoV-2 RBD [5, 18]. Interestingly, ferrets have also been observed to infect each other through direct or indirect contact [5, 19, 20], suggesting that ferrets could also be infected by contact with an infected owner, especially since they are quite sociable animals. However, in the natural environment, it appears that although these animals are susceptible to infection, the seroprevalence rates are rather low, as demonstrated by a study conducted in Spain, in which only two out of 127 tested ferrets were seropositive [8]. In our study using a relatively small sample panel (n = 45), no SARS-CoV-2-specific antibodies were detected in any of the tested animals, even though 13 of the ferrets had direct contact (e.g., cuddling) with infected people for 14 days (the official quarantine period). This may indicate that ferrets have a lower susceptibility to the virus in nature than by direct inoculation with high-titre preparations under experimental conditions. Our results are consistent with those obtained by Sawatzki et al. [21], who showed that, despite direct contact with two positivepersons, no ferrets out of 29 became infected with SARS-CoV-2. The authors suggested that mutations in the viral genome may be necessary for ferret infection to occur more frequently under natural conditions. The N501T mutation in the receptor binding motif of the SARS-CoV-2 surface glycoprotein, which interacts with ACE2, provides an example. This mutation was found to be present in all isolates that were able to cause infection in ferrets, but interestingly, this mutation is not necessary to cause infection in mink [21]. Alarmingly, virus variants with this mutation are increasingly found among human isolates, which may suggest a higher risk of SARS-CoV2 infection for ferrets under natural conditions in the future, but further studies are needed to verify this hypothesis [11, 22–24].

In our study, clinical signs in a few ferrets were reported by the owners; however, it is difficult to say whether these clinical signs were related to the ongoing pandemic, especially as the exact time point of the infection of the owners and whether the clinical signs were observed in the ferrets in the same period is not known to the authors. But as every animal tested negative for SARS-CoV-2 antibodies, it is unclear whether the observed clinical signs in the period prior to sampling are related to SARS-CoV-2 infections. At the time of sampling, Poland was already past its 3rd wave of SARS-CoV-2 circulation, with delta and alpha being the dominant virus variants in August 2021 [25].

In humans, antibody levels are thought to be fairly stable for several months [26–28] and in white-tailed deer neutralizing antibodies are measurable for more than a year [29]. However, it is not known how long antibodies persist in ferrets. In cats serum antibody levels decline to the limit of detection within about only 110 days [30]. Therefore, it could be possible that the onset of the illnesses/clinical signs in the ferrets sampled in this study was a consequence of an earlier SARS-CoV-2 infection, but that on the day of sampling, antibody levels were already below the detection limit.

Although SARS-CoV-2 transmission to pet ferrets from their infected owners has been occasionally reported in circumstances of high viral circulation in the human population [8, 14, 15, 31], there is currently no indication for frequent virus transmission from human beings to ferrets. However, given the ability of SARS-CoV-2 to rapidly mutate, this species should be included in monitoring studies.

## Supplementary Information

**Additional file 1**: Detailed information about the ferrets (n=45) that were tested for antibodies against SARS-CoV-2. Gender, age, place of living, clinical signs between January 2020 and mid-2021 as reported by the animal owner and information of confirmed SARS-CoV-2 infections of the owners are given. M – male, F – female 

## Data Availability

The datasets generated are available from the corresponding author on reasonable request.

## Abbreviations

ELISA: Enzyme-linked immunoassay
SARS-CoV-2: Severe acute respiratory syndrome coronavirus 2
WHO: World Health Organization
RBD: Receptor binding domain
RT-PCR: Reverse transcription polymerase chain reaction
OD: Optical density
