# Supplementary material for: No indication for SARS-CoV-2 transmission to pet ferrets, in five cities in Poland, 2021 - antibody testing among ferrets living with owners infected with SARS-CoV-2 or free of infection
Source: Acta Vet Scand. 2023 Feb 28;65:9. doi: 10.1186/s13028-023-00672-3 (PMC9974054; doi:10.1186/s13028-023-00672-3)
Supplement: Supplementary file 1 — Additional file 1: Detailed information about the ferrets (n=45) that were tested for antibodies against SARS-CoV-2. Gender, age, place of living, clinical signs between January 2020 and mid-2021 as reported by the animal owner and information of confirmed SARS-CoV-2 infections of the owners are given. M – male, F – female [file 13028_2023_672_MOESM1_ESM.doc]

**Additional file 1**  Detailed information about the ferrets (n=45) that were tested for antibodies against SARS-CoV-2. Gender, age, place of living, clinical signs between January 2020 and mid-2021 as reported by the animal owner and information of confirmed SARS-CoV-2 infections of the owners are given. M – male, F – female

| Animal ID | Gender | Age (years) | Place of living | Clinical signs/ diseases during SARS-CoV-2 pandemic | Owner SARS-CoV-2 confirmed | Result of the RBD ELISA (corr. OD / status) |
| --- | --- | --- | --- | --- | --- | --- |
| 1 | M | 5 | Warsaw | Pneumonia | Yes | -0.12 / negative |
| 2 | F | 3 | Warsaw | None | Yes | 0.05 / negative |
| 3 | F | 3 | Warsaw | None | Yes | 0.00 / negative |
| 4 | F | 4 | Warsaw | None | No | -0.01 / negative |
| 5 | M | 5 | Warsaw | None | Yes | -0.02 / negative |
| 6 | M | 6 | Warsaw | None | Yes | -0.02 / negative |
| 7 | M | 3 | Warsaw | None | No | 0.06 / negative |
| 8 | F | 6 | Warsaw | None | No | 0.15 / negative |
| 9 | F | 3 | Warsaw | None | No | 0.01 / negative |
| 10 | M | 4 | Warsaw | None | No | -0.03 / negative |
| 11 | M | 4 | Warsaw | None | No | 0.01 / negative |
| 12 | F | 5 | Warsaw | None | No | -0.00 / negative |
| 13 | M | 5 | Warsaw | None | No | -0.01 / negative |
| 14 | M | 5 | Warsaw | None | Yes | 0.01 / negative |
| 15 | M | 5 | Warsaw | Splenomegaly | Yes | 0.02 / negative |
| 16 | M | 7 | Warsaw | Bronchitis | No | 0.08 / negative |
| 17 | F | 3 | Wroclaw | None | No | 0.00 / negative |
| 18 | M | 6 | Wroclaw | None | No | -0.01 / negative |
| 19 | F | 4 | Wroclaw | None | Yes | 0.01 / negative |
| 20 | M | 4 | Wroclaw | None | Yes | 0.18 / negative |
| 21 | M | 6 | Wroclaw | None | Yes | 0.05 / negative |
| 22 | F | 3 | Wroclaw | None | No | 0.02 / negative |
| 23 | F | 8 | Świdnica | None | No | -0.15 / negative |
| 24 | F | 7 | Świdnica | None | Yes | -0.02 / negative |
| 25 | M | 3 | Świdnica | None | No | -0.05 / negative |
| 26 | F | 5 | Wałbrzych | None | No | -0.15 / negative |
| 27 | F | 3 | Świdnica | None | No | 0.02 / negative |
| 28 | M | 4 | Świdnica | None | No | -0.01 / negative |
| 29 | M | 4 | Świdnica | None | No | -0.07 / negative |
| 30 | F | 6 | Świdnica | None | No | -0.01 / negative |
| 31 | F | 8 | Świdnica | Chronic renal failure | No | -0.04 / negative |
| 32 | F | 4 | Świdnica | None | No | 0.00 / negative |
| 33 | M | 3 | Świdnica | None | No | -0.02 / negative |
| 34 | F | 3 | Świdnica | None | No | -0.28 / negative |
| 35 | F | 2 | Świdnica | None | No | -0.07 / negative |
| 36 | F | 4 | Świdnica | None | No | -0.05 / negative |
| 37 | M | 5 | Świdnica | None | No | -0.01 / negative |
| 38 | M | 5 | Świdnica | None | No | -0.24 / negative |
| 39 | F | 5 | Gdansk | Sneezing | Yes | 0.04 / negative |
| 40 | F | 6 | Gdansk | cancer | No | 0.03 / negative |
| 41 | M | 6 | Gdansk | None | No | 0.00 / negative |
| 42 | M | 5 | Gdansk | None | No | 0.05 / negative |
| 43 | M | 5 | Gdansk | Sneezing | Yes | 0.07 / negative |
| 44 | F | 7 | Gdansk | None | No | 0.09 / negative |
| 45 | F | 6 | Gdansk | None | No | 0.02 / negative |
